# Synchronization Dynamics in Non-Normal Networks: The Trade-Off for Optimality

**DOI:** 10.3390/e23010036

**Published:** 2020-12-29

**Authors:** Riccardo Muolo, Timoteo Carletti, James P. Gleeson, Malbor Asllani

**Affiliations:** 1 Department of Mathematics and naXys, Namur Institute for Complex Systems, University of Namur, rue Grafé 2, 5000 Namur, Belgium; timoteo.carletti@unamur.be (T.C.); Malbor.Asllani@ul.ie (M.A.); 2 MACSI, Department of Mathematics and Statistics, University of Limerick, V94 T9PX Limerick, Ireland; james.gleeson@ul.ie

**Keywords:** non-normal networks, synchronization, optimal networks, master stability function

## Abstract

Synchronization is an important behavior that characterizes many natural and human made systems that are composed by several interacting units. It can be found in a broad spectrum of applications, ranging from neuroscience to power-grids, to mention a few. Such systems synchronize because of the complex set of coupling they exhibit, with the latter being modeled by complex networks. The dynamical behavior of the system and the topology of the underlying network are strongly intertwined, raising the question of the optimal architecture that makes synchronization robust. The Master Stability Function (MSF) has been proposed and extensively studied as a generic framework for tackling synchronization problems. Using this method, it has been shown that, for a class of models, synchronization in strongly directed networks is robust to external perturbations. Recent findings indicate that many real-world networks are strongly directed, being potential candidates for optimal synchronization. Moreover, many empirical networks are also strongly non-normal. Inspired by this latter fact in this work, we address the role of the non-normality in the synchronization dynamics by pointing out that standard techniques, such as the MSF, may fail to predict the stability of synchronized states. We demonstrate that, due to a transient growth that is induced by the structure’s non-normality, the system might lose synchronization, contrary to the spectral prediction. These results lead to a trade-off between non-normality and directedness that should be properly considered when designing an optimal network, enhancing the robustness of synchronization.

## 1. Introduction

Systems in nature are often constituted by a large number of small parts that continuously interact with each other [1,2]. Although it might be possible to accurately know the dynamics that characterize each of the individual constituents, it is, in general, nontrivial to figure out the collective behavior of the systems as a whole that results from the individual/local interactions. A relevant example is provided by a system that is composed by an ensemble of coupled non-linear oscillators, which behave in unison, being driven by the non-local interaction; the system is said to be synchronized [2,3]. Synchronization has been extensively studied in snetworkcience as a paradigm of dynamical processes on a complex network, mainly due to the essential role of the coupling topology in the collective dynamics [1]. Its generic formulation allowed for researchers to use it to model several applications, ranging from biology, e.g., neurons firing in synchrony, to engineering, e.g., power grids [4]. The ubiquity of synchronization in many natural or artificial systems has naturally raised questions regarding the stability and robustness of synchronized states [5,6,7,8]. In their seminal work, Pecora & Caroll [9] introduced a method, known as Master Stability Function (MSF), in order to help understand the role that the topology of interactions has on system stability. Assuming a diffusive-like coupling among the oscillators, the MSF relates the stability of the synchronous state to the nontrivial spectrum of the (network) Laplace matrix; in particular, it has been proven that the latter should lie in the region where the Lyapunov exponent that characterizes the MSF takes negative values [2,10]. For a family of models (e.g., Rössler, Lorenz, etc.), whose stable part of the MSF has a continuous interval where the (real part of the) Laplacian eigenvalues can lie, it has been proven that they maximize their stability once the coupling network satisfies particular structural properties. Such optimal networks should be directed, spanning trees and without loops [5,6]. These networks have the peculiarity of possessing a degenerate spectrum of the Laplacian matrix and laying in the stability domain that is provided by the MSF. The Laplacian degeneracy is also often associated with a real spectrum or with considerably low imaginary parts when compared to the real ones [11,12].

The vast interest in complex networks in recent years has also provided an abundance of data on empirical networked systems that initiated a large study of their structural properties [1]. From this perspective, it has been recently shown that many real networks are strongly directed, namely they possess a highly asymmetric adjacency matrix [13]. Most of these networks present an extremely hierarchical, almost-DAG (Directed Acyclic Graph), structure. This property potentially makes the real networks suitable candidates for optimally synchronized dynamical systems that are defined on top of them. Another aspect that is unavoidably associated with the high asymmetry of real networks is their non-normality [13], namely their adjacency matrix A satisfies the condition $AA^{T}\neq A^{T}A$ [12]. The non-normality can be critical for the dynamics of networked systems [13,14,15,16,17,18]. In fact, in the non-normal dynamics regime, a finite perturbation regarding a stable state can undergo a transient instability [12], which, because of the nonlinearities, could never be reabsorbed [13,14]. The effect of non-normality in dynamical systems has been studied in several contexts, such as hydrodynamics [19], ecosystems stability [20], pattern formation [21], chemical reactions [22], etc. However, it is only recently that the ubiquity of non-normal networks and the related dynamics have been put to the fore [13,14,15,16,17,18]. In this paper, we will elaborate on these lines showing the impact of non-normality on the stability of a synchronous state. We first show that a strongly non-normal network has, in general, a spectrum that is very close to a real one, and that this, in principle, should imply a larger domain of parameters for which stability occurs, for systems with a generic shaped MSF. For illustration purposes, we will consider the Brusselator model [23,24], a two-species system with a discontinuous interval of stability in the MSF representation. We will also examine the limiting cases of our analysis to two simple network models [25], namely a (normal) bidirected circulant network and a (non-normal) chain, both with tunable edge weights in such a way to allow for a continuous adjustment, respectively, of the directedness and non-normality. In the Appendix A, we will extend such results to a family of non-normal random networks thus showing the generality of such behavior.

The MSF relies on the computation of the (real part of the maximum) Lyapunov exponent and, thus, in the case of time-dependent systems, it does not possess the full predictability power that it has in the autonomous case (fixed point in/stability). For this reason, we will use a homogenization method, whose validity is limited to a specific region of the model parameters, allowing for us to transform the linearized periodic case problem into a time-independent one [26]. This way, we remap our problem to an identical one that was studied in the context of pattern formation in directed networks where spectral techniques provide significant insight [25,27]. Such an approach allows for us on one side to assess the quantitative evaluation of the role of the imaginary part of the Laplacian spectrum in the stability problem. On the other, it permits the use of numerical methods, such as the pseudo-spectrum [12] in the study of the non-normal dynamics. To the best of our knowledge, this is the first attempt to use such techniques in the framework of time-varying systems, with the theory of non-normal dynamical systems being limited so far to autonomous systems [12]. As expected, the non-normality plays against the stability of the synchronized ensemble of oscillators. Furthermore, a high non-normality translates to a high spectral degeneracy, which brings a large pseudo-spectrum, indicating a high sensibility towards the instability.

Clearly, the directionality and non-normality stand on two parallel tracks regarding the stability of synchronized states and their robustness. The results that we present here show that the previously optimal networks are not practically as good as was thought, since the synchronization dynamics are frail to small external perturbations. In fact, their highly directed structure amplifies such perturbations and eventually causes the underlying system to desynchronize. Furthermore, such an outcome bypasses the picture that is described by the classical spectral analysis. As a conclusion of our work, we show that the most optimal design should be looked at as a trade-off between a high and low directionality/non-normality. Such a choice should depend either on the magnitude of perturbation or the ratio directed vs. non-normal of the network structure.

## 2. Optimal Synchronization: Directed vs. Non-Normal Networks

We consider a network that is constituted of *N* nodes (e.g., the idealized representation of a cell), and we assume a metapopulation framework, where the species dynamics inside each node is described by the *Brusselator* model, a portmanteau term for Brussels and oscillator. It has been initially introduced by Prigogine & Nicolis in order to capture the autocatalytic oscillation [23] phenomenon, which results from a Hopf bifurcation curve in the parameter plane. This will be the framework that we will consider in the following, thus neglecting the fixed point regime. Species can migrate across nodes with a diffusion-like mechanism. In formulae, this model translates to a reaction–diffusion set of equations:

(1) $$\left\{\begin{array}{c} \frac{d\varphi_{i}}{dt}=1-\left(b+1\right)\varphi_{i}+c\varphi_{i}^{2}\psi_{i}+D_{\varphi }\sum_{j=1}^{N}L_{ij}\varphi_{j}\\ \frac{d\psi_{i}}{dt}=b\varphi_{i}-c\varphi_{i}^{2}\psi_{i}+D_{\psi }\sum_{j=1}^{N}L_{ij}\psi_{j}\;,\;\forall i=1,\cdots ,N, \end{array}\right.$$

where $\varphi_{i}$ and $\psi_{i}$ indicate the concentration of the two species per node, $D_{\varphi }$, $D_{\psi }$ are their corresponding diffusion coefficients, and *b*, *c* are the model parameters. The coupling is represented by the matrix 𝓦, whose non-negative entries $W_{ij}$ represent the strength of the edge pointing form node *j* to node *i*. The entries of the Laplacian matrix 𝓛 are given by $L_{ij}=W_{ij}-k_{i}^{in}\delta_{ij}$, where $k_{i}^{in}=\sum_{j}W_{ij}$ stands for the incoming degree of node *i*, i.e., the number of all the entering edges into node *i*. Here, we want to emphasize that many other coupling operators are also possible; nevertheless, most of them will reduce at the linear level to a Laplacian involving the differences of the observable among coupled nodes [2], i.e., $\sum_{j=1}^{N}L_{ij}x_{j}=\sum_{j=1}^{N}W_{ij}\left(x_{j}-x_{i}\right)$. This form ensures that the coupling is only in action when the observables assume different values in two coupled nodes.

The reason for choosing such a model, as mentioned earlier, is mainly due to the discontinuous interval of the stability domain that is provided by the MSF of the problem. In order to proceed with the stability analysis, we first need to identify the homogeneous periodic solution, $\varphi^{*}\left(t\right)$ and $\psi^{*}\left(t\right)$, hereby called the *synchronized manifold* and then to linearize the system around this. Let us introduce the perturbations for the *i*–th node by $\delta \varphi_{i}$ and $\delta \psi_{i}$, and then the linearized equations describing their evolution are given by:

(2) $$\begin{array}{ccc} \frac{d(\delta \varphi_{i})}{dt} & = & \left[f_{\varphi_{i}}\delta_{ij}+D_{\varphi }\sum_{j=1}^{N}L_{ij}\right]\delta \varphi_{j}+f_{\psi_{i}}\delta \psi_{i}\\ \frac{d(\delta \psi_{i})}{dt} & = & g_{\varphi_{i}}\delta \varphi_{i}+\left[g_{\psi_{i}}\delta_{ij}+D_{\psi }\sum_{j=1}^{N}L_{ij}\right]\delta \psi_{j}\;\\ \forall i= & 1 & ,\cdots ,N, \end{array}$$

where the partial derivatives are given by $f_{\varphi_{i}}=-\left(b+1\right)+2c\varphi^{*}\left(t\right)\psi^{*}\left(t\right)$, $f_{\psi_{i}}=c\varphi^{*}{\left(t\right)}^{2}$, $g_{\varphi_{i}}=b-2c\varphi^{*}\left(t\right)\psi^{*}\left(t\right)$, and $g_{\psi_{i}}=-c\varphi^{*}{\left(t\right)}^{2}$. Notice that the partial derivatives of the reaction part are evaluated on the synchronized manifold. This translates into a time-dependent Jacobian matrix, due to the periodicity of the solutions and, thus, to a non-autonomous linear system. In order to make a step forward, let us introduce the following compact notation; let $x={\left(\delta \varphi_{1},\ldots ,\delta \varphi_{N},\delta \psi_{1},\ldots ,\delta \psi_{N}\right)}^{T}$ be the 2N-dimensional perturbations vector, 𝓓 the diagonal diffusion coefficients matrix, and 𝓙(t) the time-dependent Jacobian matrix; hence, Equation (2) can be rewritten as

(3) $$\dot{x}=\left(𝓙(t)+𝓓⊙𝓛\right)x\;,$$

where ⊙ is the coordinatewise multiplication operator. Subsequently, we proceed by diagonalizing the linearized system while using the basis of eigenvectors of the network Laplace operator 𝓛. Notice that this is not always possible, because the Laplacian matrix of directed networks might not have linearly independent eigenvectors. We will assume such a basis to exist for the time being, and we will consider such an issue again when discussing the non-normal case. Denoting, by ξ, the transformed perturbations vector, Equation (3) becomes

(4) $$\dot{\xi }=\left(𝓙(t)+𝓓⊙\Lambda \right)\xi ,$$

where Λ denotes the diagonal matrix of the Laplacian eigenvalues. The (real part of the) largest Lyapunov exponent of Equation (4), which is known in the literature as the Master Stability Function [1,2,9,10], is thus a function of the eigenvalues Λ. Let us stress that the study of the stability of a general non-autonomous system is normally not possible through the classical spectral analysis, and one, therefore, has to resort to the MSF.

**Figure 1:**
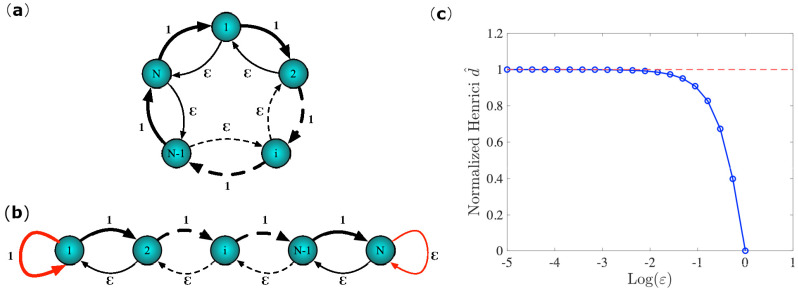
The network toy models for the case of a normal bidirectional circulant network, panel (**a**), and a non-normal bidirectional chain, panel (**b**). (**c**) Normalized Henrici’s departure from non-normality as a function of tuning parameter ϵ for the non-normal model. We observe that, starting from 0, the network is symmetric, and the non-normality increases as the weight of the reciprocal edges decreases, taking the maximal value of non-normality in the limit when ε=0. In this case, the Laplacian spectrum is degenerated.

Before proceeding in the quest for the optimal network topological features that minimize the MSF, we will introduce two simple network models, as shown in Figure 1, for which we can tune the directionality and the non-normality acting on a single parameter. In the first case, Figure 1a, we consider a bidirectional circulant network, i.e., a network whose adjacency matrix is circulant [28], made by two types of links, one of weight 1 forming a clockwise ring and the other winding a counterclockwise ring of tunable weights ϵ. The latter can vary in the interval ε∈[0,1], exploring, in this way, the possible topologies from a fully symmetric case when ε=1 to a totally mono-directed network when ε=0. Because such a network is circulant, the adjacency matrix will be normal, a property that is inherited by the Laplace operator. On the contrary, if we remove two reciprocal links, respectively, of weights 1 and ε, we, instead, obtain a non-normal network, as depicted in Figure 1b. In this case, the adjacency matrix is non-normal [12], a feature that is also reflected on the Laplacian matrix. Even in this case, we can tune the non-normality by varying the ε parameter in the unitary interval, as for the previous case, this can be appreciated from the results that are shown in Figure 1c, where we report the normalized Henrici index, a well-known proxy of non-normality, as a function of ε. The main advantage of using the above network models is the existence of a basis of eigenvectors for the Laplacian matrix. In the first network model, this is due to the normality of the graph Laplacian, while, in the second one, it is because of the tridiagonal form of the coupling operator [29]. This property is essential for the applicability of the MSF analysis, which is impossible otherwise.

## 3. The Case of Normal Directed Networks

**Figure 2:**
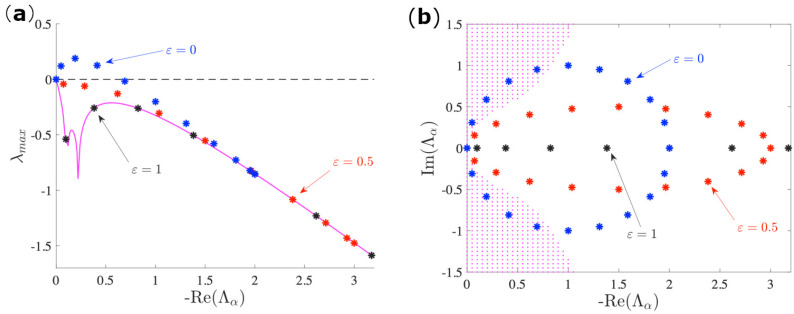
(**a**) Master Stability Function (MSF) for the Brusselator model with b=2.5, c=1 (limit cycle regime), $D_{\varphi }=0.7$, $D_{\psi }=5$ on a circulant network of 20 node; $\Lambda_{\alpha }$ indicates the the Laplacian’s eigenvalues, of which we plot only the real part. The magenta curve is obtained by integrating Equation (4) where now $\Lambda_{\alpha }$ is assumed to be a variable that takes values in a continuous non-positive interval. The symbols instead correspond to values of the MSF for discrete values of the Laplacian spectrum $\Lambda_{\alpha }$. In this setting the system should remain stable after a small perturbation: in fact, when the network is symmetric (ε=0), the discrete MSF (black dots) lies on the continuous one (magenta line); however, when we introduce an asymmetry in the topology as ε decreases (red and blue dots), the MSF reaches the instability region, and the system loses synchronization. (**b**) The equivalent representation in the complex domain, where the instability region (shaded magenta) is the one where the Lyapunov exponent is positive, assuming once again −Re$\left(\Lambda_{\alpha }\right),\pm$ Im$\left(\Lambda_{\alpha }\right)$ to be replaced by variables that take continuous values. The symbols denote the discrete Laplacian spectrum. The synchronized state is lost for the network topology with at least one eigenvalue that lies in the instability region.

We start by considering the bidirected circular network and studying the linear stability of the synchronized state while using the MSF analysis. The results that are shown in Figure 2a indicate that the network topology increasingly contrasts the stability of the synchronous manifold when the directionality increases. In fact, when the MSF computed for the directed network is compared to the symmetric case used as reference line, which lies in the magenta curve (constructed for a real continuous interval of non-positive values of $\Lambda_{\alpha }$), we can always observe larger values, which, moreover, increase as ε decreases (for the same fixed Laplacian eigenvalue). Because of the circulant property of the Laplace matrix, its spectrum can be explicitly computed [25] $\Lambda_{\alpha }=1+\varepsilon +\left(1+\varepsilon \right)\cos\left(2\alpha \pi /N\right)+i\left(1-\varepsilon \right)\sin\left(2\alpha \pi /N\right)$. One can easily notice that, for ε=0, the spectrum distributes uniformly onto the unitary circle centered at (1,0), as also shown in Figure 2b in blue stars, where the instability region in the plane [−Re$\left(\Lambda_{\alpha }\right)$, ± Im$\left(\Lambda_{\alpha }\right)]$, where the Lyapunov exponent that takes positive values is shown in magenta. On the other side, when ε=1, the network turns symmetric, which makes the spectrum real.

The MSF formalism ultimately relies on the maximum Lyapunov exponent, which, despite having proved its validity in ruling out the chaotic behavior of dynamical system [3], remains grounded on numerical methods. In order to improve our analytical understanding of the problem, we proceed by transforming Equation (4) into an autonomous one, allowing, in this way, to deploy the spectral analysis tools. This method is part of the broader set of homogenization methods that aim at averaging a time-dependent system in order to obtain a time-independent one [26]. Such methods have been found to also be useful for the stability analysis of synchronized states [30,31]. The resulting autonomous version of the MSF is sometimes referred to as the dispersion relation [21]. The mathematical validity of the proposed approximation is grounded on the Magnus series expansion truncated at the first order [31]; hence, the set of model parameters for which we expect a good agreement with the original model corresponds to the case when higher-order terms are negligible. For more details, the interested reader should consult [31]. In formula, it translates to

(5) $$𝓙\left(t\right)⟶{\langle 𝓙\rangle }_{T}=\frac{1}{T}\int_{0}^{T}𝓙\left(\tau \right)d\tau \;.$$

**Figure 3:**
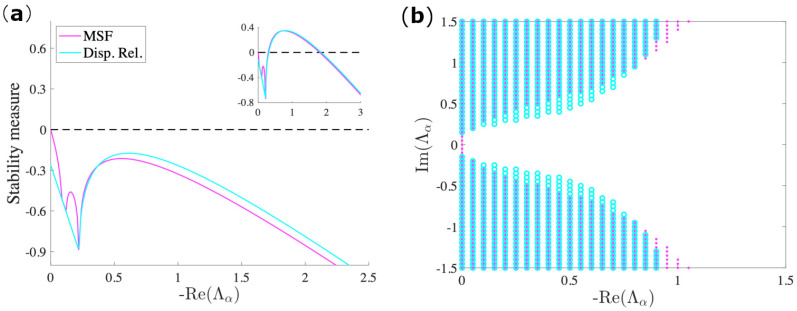
(**a**) The comparison of the MSF and dispersion relation for the Brusselator with model parameters b=2.5, c=1, $D_{\varphi }=0.7$, $D_{\psi }=5$. In magenta, we depict the MSF of the system in a limit cycle regime and cyan the dispersion relation of the averaged autonomous system. Inset: a similar comparison for a set of parameters where the instability occurs, namely b=3, c=1.8$D_{\varphi }=0.7$, $D_{\psi }=5$. Notice also the lack of continuity of the stability interval of eigenvalues. (**b**) The same representation in the complex domain (b=2.5, c=1, $D_{\varphi }=0.7$, $D_{\psi }=5$). We see that, for the chosen values of the parameters, the two approaches give an excellent agreement in predicting the instability interval.

Remarkably, as shown in Figure 3, this approximation yields qualitative results that are in excellent agreement with the original model for a specific range of parameters. An alternative to this approach is to apply a perturbative expansion near the bifurcation point, obtaining, in this way, the time-independent Ginzburg–Landau normal form [32]. However, the effectiveness of the latter method is exclusively limited to parameter values that are very close to the stability threshold. In this sense, our approach is more general, both from allowing a larger set of parameters where the method remains valid, and at the same time, it is independent of the choice of the model compared to previous works [33]. The passage to an autonomous system is also essential in explaining the effect of the imaginary part of the Laplacian eigenvalues in the newly obtained stability function, the dispersion relation. It has been rigorously shown in [25,27] that the dispersion relation increases proportionally to the magnitude of the imaginary part of the spectrum. We already observed similar results for the case of the MSF that is presented in Figure 2. We can, in this way, conclude that the averaging method sheds light on the role of the directed topology in the destabilization of a synchronized regime.

### The Case of Non-Normal Directed Networks

**Figure 4:**
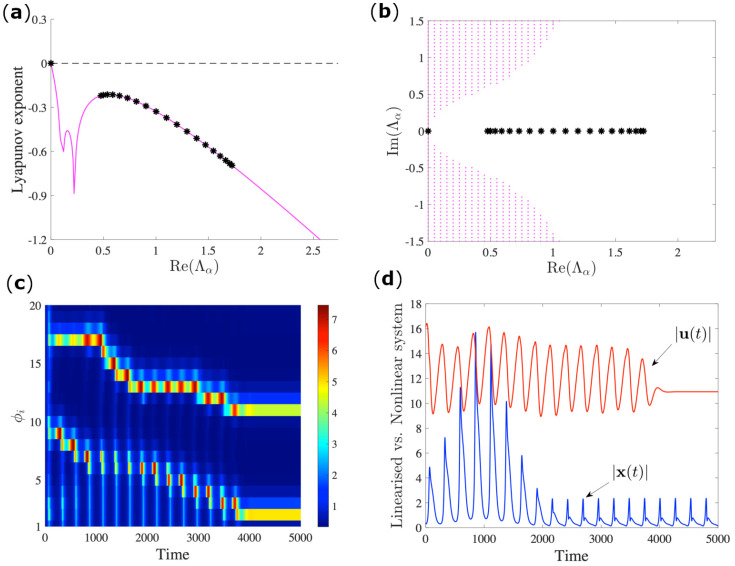
Desynchronization in a non-normal network. The parameters for the Brusselator model are as follows b=2.5, c=1, $D_{\varphi }=0.7$, $D_{\psi }=5$ on the (directed chain) non-normal network of 20 nodes with ε=0.1 of Figure 1b. As can be observed from panels (**a**,**b**), respectively, for the MSF and the stability region, the set of parameters is such that the MSF is neatly stable. The magenta curve of panel (**a**) and the instability region (magenta dots) of panel (**b**) are obtained with the same numerical procedure that is described in Figure 2. Nevertheless, the instability occurs, as shown by the pattern evolution in panel (**c**) at odds with the outcome that would have been expected from the symmetrized version. Such a result is strong evidence of the role of the network non-normality in the nonlinear dynamics of the system under investigation. The mechanism that drives the instability in the non-normal linearized regime manifests in the transition growth of the perturbations vector x(t) Equation (3), the blue curve in panel (**d**), before the system relaxes to the oscillatory state of the equilibrium. Such growth might transform into a permanent instability for the nonlinear system $u\left(t\right)=\left[\varphi (t),\psi (t)\right]$, red curve.

The analysis that was performed in the previous section is based on the study of the linearized system; in some cases, however, such an analysis is not sufficient for understanding the outcome of the nonlinear system. In Figure 4, we again consider the MSF that is computed for the directed chain previously introduced (panel *b*) of Figure 1. From Figure 4b, one might naively conclude that the system will synchronize, since the MSF is non-positive for all values of −Re $\left(\Lambda_{\alpha }\right)$. Moreover, the spectrum is completely real (see panel *b*) and, thus, there cannot be any contribution from the imaginary part of the spectrum. However, a direct inspection of the orbit behavior (panel *c*) that was obtained by numerically integrating the network of coupled Brusselator oscillators clearly shows that the system does not synchronize. This diversity of behavior is related to the non-normal property of the considered network; indeed, it has been recently proved that such a structural property can strongly alter the asymptotic behavior of networked systems [34]. In a linear regime, a finite perturbation regarding a stable equilibrium goes through a transient amplification (blue curve in Figure 4d) that is proportional to the level of non-normality before it is eventually reabsorbed in the steady state [12], while, in the full non-linear system, the finite perturbation could persist indefinitely (red curve in Figure 4d). Up to now, this analysis has been limited to the case of autonomous systems; in this paper, for the first time we extend it to the periodic time-dependent case, making use of the homogenization process. This explains the permanent instability, as shown in Figure 4, causing the loss of stability for the synchronized state.

**Figure 5:**
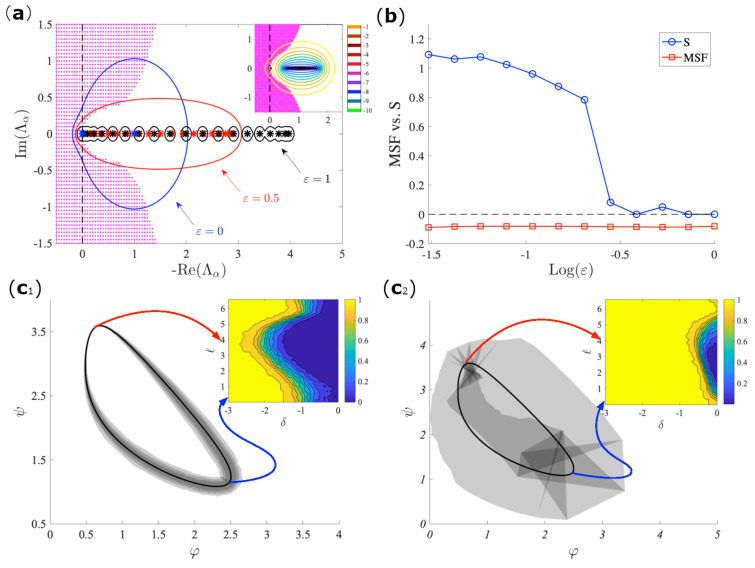
(**a**) The pseudo-spectral description of the stability of the directed in a non-normal network. The parameters for the Brusselator model are as follows b=2.5, c=1, $D_{\varphi }=0.7$, chain of 20 nodes for the Brusselator model with b=2.5, c=1.12, $D_{\varphi }=0.7$, $D_{\psi }=5$, and an initial condition perturbation of the average magnitude δ=0.1. We show the pseudo-spectra for three different values of the control parameter ε for the chain network, emphasizing the considerably large difference between the pseudo-spectra regions and the spectrum of the Laplacian matrix. Inset: the pseudo-spectra for many other values of the perturbation magnitude δ for the chain with ε=0.1. Notice that, although the eigenvalues do not lie inside the instability region due to the lack of an imaginary part, the pseudo-spectra might do. (**b**) The comparison between the expected outcome, as predicted from the MSF, and the actual outcome, as measured by the standard deviation of the desynchronized pattern. The stability basin (shaded gray) projected onto the limit cycle plane for the non-normal case, panel (**c**${}_{1}$) and the symmetrized (normal) one, panel (**c**${}_{2}$), calculated over 300 different initial conditions (of the same averaged magnitude), and a perturbation whose maximum magnitude varies from $10^{-3}$ to 1. Inset: in the *y*-axis, we plot the points of limit cycle that we perturb and, in the *x*-axis, the magnitude of the perturbation; the colormap gives the fraction of orbits that conserve the synchronized regime. It can be clearly noticed that the attraction basin for the non-normal network is strongly reduced, although not at the same amount when compared to where the perturbation occurs.

The non-normal dynamics study cannot be straightforwardly tackled with the analytical methods of the local stability, mostly because the instability occurs in a highly nonlinear regime. Such condition require a global analysis that can be obtained while using the numerical technique that is based on a spectral perturbation concept that is known as the pseudo-spectrum. For a given matrix A, the latter is defined as $\sigma \left(A_{\delta }\right)=\sigma \left(A+\Delta \right),$ for all ||Δ||≤δ, for where σ(·) represents the spectrum and ||·|| a given norm. The package EigTool [35] allows for us to compute and draw in the complex plane the level curves of the pseudo-spectrum for a given value of δ. Although the pseudo-spectrum is not sufficient to fully explain the system behavior, it is certainly of great utility in estimating the role of non-normality in the dynamics outcomes. In particular, in panel (*a*) of Figure 5, we report level curves of the pseudo-spectrum for three different values of the parameter ε representing the reciprocal links of the directed chain. Notice that, by increasing the non-normality of the toy network, the pseudo-spectrum will also increase the chances of intersection with the instability region. In panel (*b*) of Figure 5, we have shown a comparison between a proxy of the presence of a synchronized state, i.e., the standard deviation *S* [36] of the asymptotic orbit behavior and the MSF demonstrating a clear different behavior. For all of the considered values of ε, the MSF is always negative, which suggests a stable synchronized state; on the other hand, *S* becomes positive and large for small enough ε, testifying a loss of synchronization. The dependence on the different values of the initial conditions is further shown in panels ($c_{1}$) and ($c_{2}$). As expected, the instability is more probable for both larger values of non-normality and magnitude of the initial conditions. In particular, it can be observed that the synchronization basin of attraction is strongly reduced for the non-normal network as compared to the normal one and, moreover, its width varies along the limit cycle, which implies that desynchronization will also depend on the point at which the perturbation starts.

## 4. Conclusions

In this paper, we have studied the quest for the optimal conditions ensuring the stability of synchronization dynamics in directed networks. Such conditions determine the design of a networked system that makes the synchronization regime as robust as possible. Previous results have proven that a strictly directed topology is necessary for the synchronized state’s robustness. Based on the well-known Master Stability Function, it has been shown that directed tree-like networks are optimal for models with a discontinuous interval of the Laplacian spectrum in the stability range of MSF. Here, we have extended such results, proving that they are generally independent of the dynamic model. While using an averaging procedure, we transformed the problem from a time-dependent (non-autonomous) to a time-invariant (autonomous) one. This method allows for proving that networks whose Laplacian matrix exhibits a spectrum that lacks an imaginary part are the most optimal. In general, the loss of synchronization increases with the magnitude of the imaginary part of the spectrum. However, our primary focus has been on the effect that the non-normality, a ubiquous feature of many real-world networks, as recent findings show [13], has on the collective dynamics of synchronization. This latter feature can play a very important role in the linear dynamics influencing the local stability of the synchronized state through a strong transient amplification of the perturbations. We have extended the idea of non-normal dynamics to the case of non-autonomous synchronization dynamics, revealing how network non-normality can drive the system to instability, thus increasing the understanding of synchronization in complex networks. We have also numerically quantified the effect of non-normality in driving the instability through the pseudo-spectrum technique. Although, throughout this paper, for purely pedagogical reasons, we have illustrated our results for a toy network model with tuneable non-normality, we have also shown (see the Appendix A) that such results extend to a general case of non-normal random networks. In conclusion, we have analytically and numerically demonstrated that there is no compelling recipe for optimal network architecture in order to conserve the synchronized state, but rather a trade-off between the network directedness and its non-normality. Our results clearly show that networks previously thought to be optimal regarding synchronization are not such, but, on the contrary, the stability of the associated synchronous solution is quite fragile to small perturbations, which makes their role in the synchronization dynamics apparently different from what was previously intuited in the literature [5,6]. Additionally, the non-normality makes standard techniques, such as the Master Stability Function, fail by a large amount. We are aware that the interesting outcomes of the interaction of structural non-normality networks with the fascinating synchronization phenomenon require deeper and further investigation (e.g., synchronization basin). In this sense, we hope that we can initiate a new direction of research of the synchronization problem with our work through this paper.

## Figures and Tables

**Figure A1:**
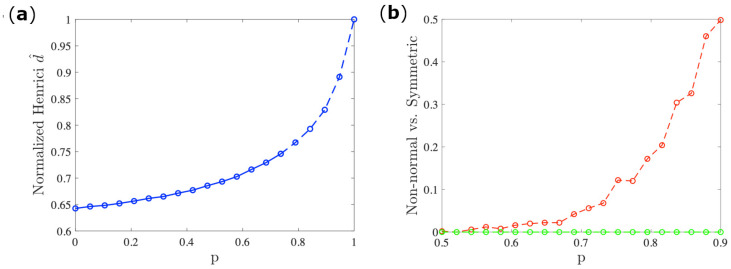
(**a**) The normalized Henrici departure from non-normality $\hat{d}$ vs. the control parameter *p* averaged over 500 independent realizations of networks generated with the algorithm described in the text for 50 nodes, and $p_{0}=0.2$. We can observe that the non-normality of the ensemble of networks increases with the non-normality. For a given threshold of the control parameter *p*, the networks become degenerate (depicted in dashed line). (**b**) The fraction of the outcomes of integrating the Brusselator model when the synchronization is lost averaged over 500 different initial conditions (red curve). The parameters are b=5, c=1.6, $D_{\varphi }=0.6$ and $D_{\psi }=4.5$, and the networks generated as in the left panel. For comparison, we have also shown the outcomes of the symmetrized network (green curve), where the synchronization is conserved in any case.

## Data Availability

No applicable.

## Appendix A

In this section, we extend our results of the main text to a family of random networks with a tuneable level of non-normality and systematically study the loss of synchronization in relation to the control parameter. We initially start with an (unweighted) directed Erdős-Rényi [37] with a fixed number of nodes *N* and a probability $p_{0}$ of having a directed edge from node *i* to node *j*. Notice that such Erdős-Rényi networks, being directed, will be automatically non-normal, and their non-normality will depend on the density of the edges and the eventual hierarchy of the network structure [13]. Our primary step for generating random networks with tuneable non-normality is to increase the network directionality, more precisely we remove entries form the lower triangular part of the adjacency matrix; this corresponds to the removal of links at random with probability *p*. The latter will be our control parameter. As can be observed from Figure A1, panel (*a*), the non-normality of the network, measured with the normalized Henrici index, monotonically increases for increasing values of the control parameter *p*.

To emphasize the consequence of the increasing non-normality in the stability of the synchronized regime, we systematically investigate the behavior of the system of coupled Brusselator oscillators [38]. As shown in Figure A1, the fraction of simulations for which the system does not synchronize when the random network becomes more non-normal increases (red curve), compared to the symmetrized counterpart (green curve) where synchronization is always achieved. This is thus the same qualitative result and prediction presented in the main text for the two simple networks. Furthermore, for values of the control parameter p≳0.75, the networks’ Laplace spectrum becomes degenerate, and the Master Stability Function approach cannot be used in its simplified form due to the lack of an eigenvector basis. Nevertheless, the fraction of cases when the system desynchronizes keeps increasing as in the case of non-degenerate spectra.

As a conclusion, we can state that the results we have shown in this paper are valid more generally, beyond the model used for the generation of the synthetic networks and directly related to the amount of the non-normality of the underlying networked structure.

References and Notes
